# Daily television exposure, parent conversation during shared television viewing and socioeconomic status: Associations with curiosity at kindergarten

**DOI:** 10.1371/journal.pone.0258572

**Published:** 2021-10-28

**Authors:** Prachi E. Shah, Kathy Hirsh-Pasek, Todd B. Kashdan, Kristen Harrison, Katherine Rosenblum, Heidi M. Weeks, Priya Singh, Niko Kaciroti

**Affiliations:** 1 Division of Developmental Behavioral Pediatrics, Department of Pediatrics, University of Michigan Medical School, Ann Arbor, MI, United States of America; 2 Department of Psychiatry, University of Michigan Medical School, Ann Arbor, MI, United States of America; 3 Department of Psychology, Temple University, Philadelphia, PA, United States of America; 4 Department of Psychology and Center for the Advancement of Well-Being, George Mason University, Fairfax, VA, United States of America; 5 Department of Communication and Media, University of Michigan, Ann Arbor, MI, United States of America; 6 Department of Nutritional Sciences, School of Public Health, University of Michigan, Ann Arbor, MI, United States of America; 7 Department of Pediatrics, University of Cincinnati Medical School, Cincinnati, OH, United States of America; 8 Department of Biostatistics, School of Public Health, University of Michigan, Ann Arbor, MI, United States of America; Williams College, UNITED STATES

## Abstract

**Objective:**

To examine the main and interactive effects of the amount of daily television exposure and frequency of parent conversation during shared television viewing on parent ratings of curiosity at kindergarten, and to test for moderation by socioeconomic status (SES).

**Study design:**

Sample included 5100 children from the Early Childhood Longitudinal Study, Birth Cohort. Hours of daily television exposure and frequency of parent screen-time conversation were assessed from a parent interview at preschool, and the outcome of early childhood curiosity was derived from a child behavior questionnaire at kindergarten. Multivariate linear regression examined the main and interactive effects of television exposure and parent screen-time conversation on kindergarten curiosity and tested for moderation by SES.

**Results:**

In adjusted models, greater number of hours of daily television viewing at preschool was associated with lower curiosity at kindergarten (B = -0.14, p = .008). More frequent parent conversation during shared screen-time was associated with higher parent-reported curiosity at kindergarten with evidence of moderation by SES. The magnitude of association between frequency of parent conversation during television viewing and curiosity was greater for children from low SES environments, compared to children from high SES environments: (SES ≤ median): B = 0.29, p < .001; (SES > median): B = 0.11, p < .001.

**Conclusions:**

Higher curiosity at kindergarten was associated with greater frequency of parent conversation during shared television viewing, with a greater magnitude of association in low-SES families. While the study could not include measures of television program content, digital media use and non-screen time conversation, our results suggest the importance of parent conversation to promote early childhood curiosity, especially for children with socioeconomic disadvantage.

## Introduction

Curiosity, an important foundation for scientific innovation [1], is characterized by the drive to seek out new information [2], desire to explore [3], and joy in learning [4, 5]. Higher curiosity has been associated with numerous adaptive outcomes in childhood including more robust word acquisition [6], enhanced learning and exploration [7] and higher academic achievement [8, 9], highlighting the potential importance of fostering curiosity from an early age. Our previous work found a positive association between higher curiosity and higher academic achievement, with a greater magnitude of benefit for children with socioeconomic disadvantage [10], raising the possibility that promoting curiosity in young children may be one way to mitigate the achievement gap associated with poverty [11]. To foster curiosity in early childhood, it is necessary to consider the modifiable contexts that may promote or inhibit its expression.

One potential modifiable factor associated with differences in early childhood outcomes is the amount of daily television exposure. While there is an increasing interest in the role of digital media exposure on child development, televisions are in 98% of all homes, and television viewing remains the dominant screen activity of young children, accounting for 72% of all screen time [12], making television exposure a relevant developmental context in young children. Children are exposed to an average of 1–4 hours of television per day [13, 14], with higher exposure in children who are economically disadvantaged [15, 16]. In previous screen-time research with infants, toddlers and preschoolers, more television exposure has been associated with impaired self- regulation [17, 18], lower language outcomes [19, 20], and lower cognitive development [21, 22], however, association with curiosity has not been examined, and is a gap in the literature. Screen media exposure, including television, can displace exploratory activities such as play and parent-child interactions [23] that are thought to be necessary for the cultivation of curiosity [24]. We therefore sought to test the hypothesis that higher daily television exposure would be associated with lower curiosity (Hypothesis 1). We also considered that the association between the amount of television exposure on early childhood curiosity may be attenuated in children with higher SES, who may have other resources to foster curiosity, compared with low SES children. Therefore, we sought to test whether the association between higher daily television exposure and early childhood curiosity was moderated by SES, with a greater magnitude of effect seen in low SES /under-resourced families (Hypothesis 2).

In addition, because development unfolds through reciprocal interactions between children and their parents, the quality of early dyadic experiences may also play a role in fostering curiosity. Previous work has demonstrated the benefits of parent conversation during shared television viewing on language development, with more frequent conversation moderating the adverse impact of heavy television exposure [25]. Previous research has also demonstrated that parent-child conversation facilitates children’s thinking, learning and exploration (i.e., behavioral indicators of curiosity) through pedagogical exchanges [26]. As such, we hypothesized that more frequent parent conversation during shared television viewing may be associated with higher curiosity (Hypothesis 3a) and may moderate the association between higher television exposure and curiosity (Hypothesis 3b). Furthermore, because the amount and quality of language that young children hear also varies by socioeconomic status [27], (e.g., the 30-million-word-gap) [28, 29], we theorized that there may be a similar “curiosity gap” among low income children who are exposed to less conversation. We hypothesized that, consistent with a cumulative risk model [30], socioeconomic disadvantage *in combination with* less frequent parental conversation may confer an added risk for lower curiosity, with greatest effects seen in children from low SES families (Hypothesis 4). The overarching aim of this work was to identify modifiable factors in the early caregiving environment (e.g., amount of early television viewing, frequency of parent conversation) which may be important for the promotion of early childhood curiosity, and to examine whether these factors were associated with differential effects in children from under-resourced families. Results from this work will help inform anticipatory guidance to promote early childhood curiosity in at risk populations.

## Materials and methods

### Study design and sample

Data were drawn from the restricted data of the Early Childhood Longitudinal Study, Birth Cohort (ECLS-B), a nationally representative, population-based longitudinal study sponsored by the US Department of Education’s National Center for Education Statistics (NCES). The ECLS-B is based on a nationally representative probability sample of children born in the United States in 2001. Data were collected from children and their parents at age 9 months, 24-months, preschool and kindergarten timepoints, and included parent interviews, and direct and indirect child assessments across multiple settings [31]. Our sample excluded children with congenital and chromosomal abnormalities, and included children born at 22–41 weeks gestation who had kindergarten behavioral data from which we could derive a measure of curiosity. Our study utilized data from birth, 24-months, preschool and kindergarten timepoints, with a final sample of 5100 children. This study was considered exempt by the Institutional Review Board because it involved the use of a publicly available dataset with de-identified participants who could not be linked to the data.

### Measures

#### Outcomes

*Curiosity*. Because the ECLS-B did not have a measure to examine curiosity, we derived a measure of curiosity from an existing assessment of child behavior available in the dataset, which included questions from the Preschool and Kindergarten Behavioral Scales Second Edition (PKBS-2) and Social Skills Rating System (SSRS). While we were limited by the questions that were available the parent PKBS-2 questionnaire at the kindergarten timepoint, we drew from previous theoretical work and behavioral descriptions of curiosity in young children [32–38] to select question items that most closely aligned with characteristics of curiosity. While there is no single definition of curiosity [33], there are certain behavioral characteristics of curiosity that are widely accepted, including, (1) *the thirst for knowledge*, *and the drive to understand what one does not know* [34]; (2) *an exploratory drive to seek novelty* [35]; (3) *an openness to new experiences* [36]; and, in young children, (4) *innovation in exploratory play* [37, 38]. Four question items from the PKBS-2 which aligned with these characteristics of curiosity were chosen for our “curiosity factor.” The individual question items included (1) *shows eagerness to learn new things* (i.e., thirst for knowledge); (2) *likes to try new things* (i.e., drive for novelty); (3) *easily adjusts to a new situation* (i.e., openness to new experiences); and (4) *shows imagination in work and play* (i.e., innovation in exploratory play). At the kindergarten timepoint, parents were asked to report the frequency of behaviors observed in the previous 3 months on a 5-point Likert scale (1, never to 5, very often). Items were reverse coded as appropriate such that higher scores indicated more positive behaviors. A confirmatory factor analysis (CFA) was conducted to assure reliability and to calculate the appropriate loading values for deriving our curiosity factor. Standardized scoring of the curiosity factor was conducted, and good internal consistency was demonstrated (α = 0.70, M = 0.07, SD = 1.2) [10]. Individual question items, loading coefficients, and model fit indices for our curiosity factor are shown in S3 Appendix.

#### Predictors

*Hours of television viewing*. Hours of television viewing at preschool were determined from a parent questionnaire at the preschool timepoint. Parents were asked “…*about how many hours of television does [your child] watch at home per day*,” with responses ranging from 0–24 hours. Respondents who answered “N/A” to this question were not included in the analysis. Because most children (96%) reportedly watched 6 hours or fewer of television per day, the hours of daily television exposure were capped at 6+ hours, reducing the influence of a few statistical outliers.

*Parent conversation during shared television viewing*. Parent conversation during television viewing was determined from a parent questionnaire at the preschool timepoint. Parents were asked, “*In a typical week*, *when your family watches TV together*, *how often do you or another family member talk with [your child] about the TV programs*?” Responses were coded categorically as 1 = never, 2 = hardly ever, 3 = sometimes, or 4 = often. Parents were not asked to report on the amount of time a child watched television without adults, thus we were unable to control for the amount of time children watched television without adult co-viewing.

Relatedly, there was also no measure of overall (non-television) parental language for the entire sample. As such, we were unable to control for *non-screen time* parental language. To address this limitation, using a subsample of 500 parent-child dyads with available data on a structured reading task, we examined the association between parent conversation during TV viewing and parent conversation during the reading task. We found a positive association for the frequency of television-related parent conversation and elaborative parent language during the reading task characterized by use of open-ended questions (p = .008) and relating the book to the child’s experience (p = .03). Based on this subsample analysis, we considered that television-related parent conversation may also reflect the quality of the language environment in the home. For the purpose of this study, we considered the amount of parent conversation during shared television viewing to serve as a proxy for the amount of language in the caregiving environment.

#### Covariates

In our primary analyses, we included sociodemographic variables that might be associated with the amount of television viewing and curiosity. Specifically, we controlled for maternal age, race/ethnicity, marital status (married/unmarried), maternal education (< high school; high school graduate; > high school), and poverty (< 185% federal poverty line; ≥ 185% federal poverty line). The latter two variables were integrated into a composite measure of household socioeconomic status (SES) at kindergarten [31]. We also controlled for child sex, child age, the type of childcare/preschool experience (no non-parental care; relative/nonrelative home-based care; center-based care), and average number of hours of childcare/center-based care per day. Because lower developmental skills [39] and inability to delay gratification [18] have been associated with increased television exposure, additional covariates included a measure of infant development at 24-months from the Bayley Short-Form Research Edition, and parent-report of delay of gratification at 24-months (“*My child is able to wait*” dichotomized as “no/yes”). Of note, there was no measure of the content of television programming available in the dataset (i.e., educational programming vs. entertainment), so we were not able to control for television content in our analyses.

### Statistical analyses

All analyses were conducted using SAS 9.4 [40] (SAS Institute Inc., Cary, NC). Maternal and child characteristics were examined using descriptive statistics. Multivariate linear regression utilizing the SURVEYREG (SAS) procedure allowed for tests of associations between hours of daily television viewing, frequency of parent conversation during shared television viewing and kindergarten curiosity in linear and non-linear (quadratic) models, with minimal differences between the linear and quadratic models. We included covariates related to television viewing, parent conversation, and curiosity to adjust for theoretically justified confounds. For our primary analyses, in adjusted models, we tested the association between the hours of television viewing and curiosity at kindergarten (Hypothesis 1), and whether the association between hours of television viewing and curiosity was moderated by SES (Hypothesis 2). We examined whether the amount of parent conversation during shared television viewing at preschool was associated with early childhood curiosity (Hypothesis 3a), and whether the amount of parent conversation moderated the association between the amount of television viewing and early childhood curiosity (Hypothesis 3b). Finally, we examined whether the association between parent conversation during television viewing and curiosity at kindergarten was moderated by SES (i.e., our test of a cumulative risk hypothesis) (Hypothesis 4). In all our moderation analyses, we included the interaction term in the final step of the multivariate regression models. When the interaction was statistically significant (p < .05), we performed a stratified analysis of the association between the predictor and curiosity, adjusting for covariates. Because of the complex sample design, sample weights and the Jackknife method [41] were used to account for stratification, clustering and unit non-response, thereby allowing the weighted results to be generalized to the population of U.S. children born in 2001. In accord with the NCES requirements for ECLS-B data use, reported numbers were rounded to the nearest 50.

## Results

### Sample characteristics

Of the 6350 children who had behavioral (curiosity) data at kindergarten, 5100 children had television-viewing data at preschool and all covariates, which served as our analytic sample. The 5100 children in our final sample did not differ from the 1250 children who were excluded (due to missing data) on most demographic characteristics. However, children who were excluded were more likely to be non-White, have lower SES, have higher 24-month development, watched fewer hours/day of television, and attended childcare/preschool more hours per day. At the preschool timepoint, parents reported that children watched an average of 2.5 hours of television per day, and almost half of parents (49.8%) reported talking with their children “often” when viewing television together. After applying sample weights, the maternal and child characteristics were generalizable to the US population in 2001. The sample characteristics for the weighted sample are shown in Table 1. Descriptive characteristics of the amount of television viewing and parent conversation during shared television viewing are shown in Table 2.

**Table 1:** Maternal and child characteristics.

**Maternal Characteristics**	Mean, SD or Weighted %
Age (years)	27.4, 4.6
Race/ethnicity	
White/Non-Hispanic	57.2%
Black/Non-Hispanic	14.5%
Hispanic	22.9%
Asian	2.9%
Other	2.4%
Marital Status	
Married	68.0%
Unmarried	32.0%
Socioeconomic indicators calculated from measures of education and income at Kindergarten:	
Maternal Education	
Less than high school	17.8%
High school graduate	29.5%
> High School	52.8%
Below poverty threshold (<185% federal poverty line)	44.2%
At or above poverty threshold (≥185% federal poverty line)	55.8%
**Child Characteristics**	Mean, SD, or Weighted %
Gender	
Male	50.5%
Female	49.5%
Preschool-age Child Care/Preschool Experience	
Parental care only (no childcare)	19.9%
Relative/non-relative care	21.4%
Center based care (Preschool or Head Start)	58.7%
Hours per day of Child-care or Preschool (hours)	4.5, 5.9
Age at Preschool (months)	52.5 (5.5)
Age at Kindergarten (months)	68.2, 7.4
24 Month Cognitive Development (T-score)	50.1, 16.3
Ability to delay gratification at 24 months (yes)	33.7%

SOURCE: U.S. Department of Education, National Center for Education Statistics, Early Childhood Longitudinal Study, Birth Cohort. Selected years 2001–2007

**Table 2:** Descriptive characteristics of amount of television viewing and parent conversation during shared television viewing.

(Weighted %)	Total Sample	Low SES	High SES
**Hours of Television Viewing/Day**			
0 hours	1.6%	1.0%	2.3%
1 hour	35.7%	28.1%	44.5%
2 hours	31.8%	31.5%	32.1%
3 hours	13.8%	16.7%	10.4%
4 hours	6.8%	8.9%	4.3%
5 hours	4.6%	5.9%	3.1%
6+ hours	5.8%	7.9%	3.3%
**Parent Conversation During *Shared* TV Viewing**			
Often	49.8%	46.5%	53.6%
Sometimes	38.8%	42.1%	35.0%
Hardly Ever	8.6%	8.5%	8.7%
Never	2.8%	2.9%	2.7%

SOURCE: U.S. Department of Education, National Center for Education Statistics, Early Childhood Longitudinal Study, Birth Cohort. Selected years 2001–2007

### Tests of association between hours of daily television viewing and child curiosity at kindergarten (Hypothesis 1), moderation by SES (Hypothesis 2), and main and moderating effects of parent conversation during shared television viewing (Hypotheses 3a and 3b)

In adjusted models, higher daily television viewing at preschool was associated with lower curiosity at kindergarten (B = -0.14, p = .008) (Hypothesis 1, S1 Appendix). The association between the hours of daily television viewing at preschool and kindergarten curiosity was not moderated by SES (p = .22) (Hypothesis 2). In adjusted models, we also found that more frequent parent screen-time conversation was associated with higher curiosity at kindergarten (p < .001), (Hypothesis 3a, S1 Appendix), but that more frequent parent conversation did not moderate the association between the amount of television exposure and early childhood curiosity (p = .23) (Hypothesis 3b).

### Tests of association between parent conversation during shared television viewing and child curiosity at kindergarten, and moderation by socioeconomic status (Hypothesis 4)

**Fig 1:**
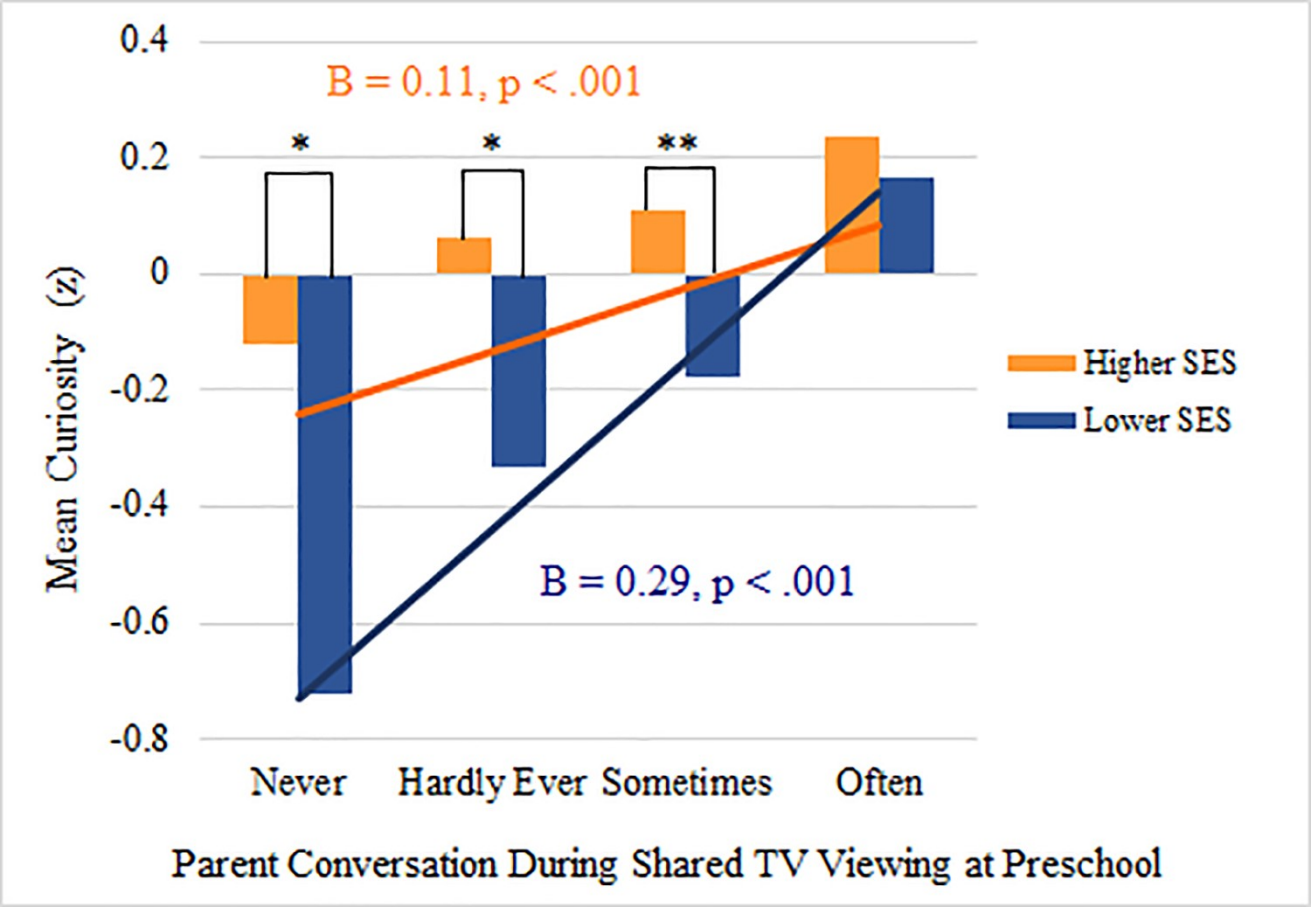
Frequency of parent conversation during shared media viewing at preschool and mean curiosity at kindergarten, stratified by higher and lower socioeconomic status (SES). B coefficients are unstandardized betas. Bars indicate parent conversation during TV viewing examined as a categorical variable. Lines indicate parent conversation during TV viewing examined as a continuous variable, to test and confirm the linear trend. *: p < .05; **: p < .01. SOURCE: U.S. Department of Education, National Center for Education Statistics, Early Childhood Longitudinal Study, Birth Cohort. Selected years 2001–2007.

We then examined whether the association between the frequency of parent screen-time conversation at preschool and kindergarten curiosity was moderated by socioeconomic status (SES) (Hypothesis 4). We found evidence of moderation by SES, (S2 Appendix), and proceeded to examine this association further by stratifying by lower SES (≤ median) and higher SES (> median), adjusting for the *a priori* covariates. We found differences in parent-reported curiosity between families from high and low levels of SES, for each category of parent conversation (*never*, *hardly ever*, *sometimes*, *often*), with stronger association among families from under-resourced environments (i.e., low SES) (Table 3). To test and confirm the linear trend between parent conversation and curiosity, *in this model only*, we then tested the association with parent conversation coded as a continuous variable (1–4). The linear trend demonstrated that the effect of more frequent parent conversation on curiosity was stronger among low SES families (B = 0.29, p < .001) compared with high SES families (B = 0.11, p < .001) (Fig 1).

**Table 3:** Adjusted associations of daily television viewing, parent conversation during shared television viewing and curiosity at kindergarten, stratified by higher and lower socioeconomic status (SES).

	Lower SES	Higher SES
Step 3 Results (with interaction, stratified by SES)	B (SE)	B (SE)
Hours of television viewing/day (linear term)	-0.16 (0.06)*	-0.14 (0.07)
Hours of television viewing/day (quadratic term)	0.02 (0.01)*	0.01 (0.01)
Parent Conversation during shared TV viewing		
Often	0.89 (0.17)***	0.36 (0.14)*
Sometimes	0.55 (0.17)**	0.23 (0.14)
Hardly ever	0.40 (0.18)*	0.18 (0.17)
Never (REF)	-----	-----

B coefficients are unstandardized betas.

*: p < .05; **: p < .01; ***: p < .001.

Analyses controlled for SES at kindergarten, maternal age, race/ethnicity, child cognitive development (24 months), child’s age, child’s sex, child’s ability to delay gratification.

SOURCE: U.S. Department of Education, National Center for Education Statistics, Early Childhood Longitudinal Study, Birth Cohort. Selected years 2001–2007.

### Frequency of parent conversation during shared television viewing and associations with characteristics of childhood curiosity

To further examine the psychometrics of our measure of curiosity and consider the value of each question item, we conducted a post hoc analysis to determine if there were specific features of childhood curiosity that were susceptible to the effects of parent conversation. We ran four models, examining the association between the frequency of parent conversation (as a continuous variable), and each curiosity question item as our outcome, adjusting for *a priori* covariates. In these models, more frequent parent conversation was positively associated with each curiosity question item, with the greatest magnitude of association demonstrated by “*shows imagination in work and play*,” (B = 0.14, p < .001) (Table 4). The relatively similar findings across items suggests that our curiosity measure tends to act as a unified construct.

**Table 4:** Association between frequency of parent conversation during shared media viewing and each curiosity question item at kindergarten.

Curiosity Question	B (SE)	p
Likes to try new things	0.11 (0.02)	< .001
Shows imagination in work and play	0.14 (0.02)	< .001
Shows eagerness to learn new things	0.11 (0.02)	< .001
Easily adjusts to a new situation	0.12 (0.02)	< .001

B coefficients are unstandardized betas.

Analyses controlled for hours of daily television viewing, maternal age, race/ethnicity, child cognitive development (24 months), SES at kindergarten, child’s age, child’s sex, child’s ability to delay gratification.

SOURCE: U.S. Department of Education, National Center for Education Statistics, Early Childhood Longitudinal Study, Birth Cohort. Selected years 2001–2007.

## Discussion

This is the first study examining associations among amount of daily television exposure, frequency of parent conversation during shared television viewing at preschool, socioeconomic status, and parent-report of curiosity at kindergarten using a nationally representative sample. In adjusted analyses, we found that higher daily television viewing at preschool had a small but significant association with *lower* curiosity at kindergarten (Hypothesis 1), but that this association was not moderated by socioeconomic status (SES) (Hypothesis 2). We found that more frequent parent conversation during shared television viewing was associated with *higher* curiosity at kindergarten (Hypothesis 3a), but that more frequent parent conversation during shared television viewing did not moderate the association between the amount of television exposure and early childhood curiosity (Hypothesis 3b). While we found an association between higher television viewing at preschool and lower parent-report curiosity at kindergarten, we were not able to include measures of the content of the television programming. Because the opportunities for conversation and scaffolding may differ if dyads are watching educational TV versus other type of programming, our inability to include the content of the television programming in our analyses (due to the constraints of the dataset) limits the interpretability of the association between the amount of television viewing and kindergarten curiosity.

We found an association between the amount of parent conversation during shared television watching at preschool and early childhood curiosity (Hypothesis 3a) with evidence of moderation by SES (Hypothesis 4). In both high and low SES families, parents who reported higher amounts of conversation also rated their children as being more curious, with a greater magnitude of association in children from under-resourced families. We have several possible explanations to account for these findings. One interpretation is that parents who report engaging in more conversation may also be more attuned to children’s expression of curiosity (e.g., children’s asking of questions, and engagement in pedagogical exchanges in conversation), and thus they also report their children as having higher curiosity. However, while greater parental conversational exchanges have been associated with more question-asking from their children [42], this explanation does not explain why the magnitude of association between the frequency of parent conversation and curiosity would be *greater* in *low SES* children. One possible explanation is that some parents may engage in frequent conversation with their children in settings other than television, but allow their children watch television alone, which may explain why there is an attenuated association between conversations during shared television watching and curiosity for higher SES parents. An alternate explanation is that while the “cumulative risks” of socioeconomic disadvantage *and* less frequent parental conversation may confer an added risk for lower curiosity [30], the same children who are *more vulnerable* to *suboptimal development* (e.g. “lower curiosity”) may also be *more susceptible* to the effects of *more stimulating caregiving* environments (e.g. more frequent parent conversation) [43]. This suggests a potential “differential susceptibility” to the quality of the caregiving environment, whereby low-SES children may reap added benefits from language-promotive environments. Prior research has demonstrated how the quality of the linguistic environment in the home (e.g. quality and quantity of language stimulation) can mitigate the effects of socioeconomic disparities (i.e., poverty) on brain structure and later language and literacy outcomes [44, 45]. Our results similarly suggest that the quality of the early linguistic environment (characterized by more frequent parent conversation during shared TV viewing) while promotive of higher curiosity in *all* children, may be *especially beneficial* to foster curiosity in children with socioeconomic disadvantage.

These findings have implications for the anticipatory guidance provided to parents. There is some evidence suggesting that children with low curiosity fail engage with their environments in ways that foster motivation, achievement, and more specifically, academic development [46]. Building on our previous work which suggested that higher curiosity can help narrow the achievement gap associated with poverty [10], our results suggest that one potential way to foster curiosity is through facilitating conversational exchanges between children and their parents around moments of shared activity, especially for children from low socioeconomic environments. This aligns with previous language-related research which demonstrates that socioeconomically disadvantaged children preferentially benefit from greater child-directed speech and conversational exchanges [27, 45, 47, 48]. Our findings also highlight the importance of parental scaffolding for child engagement and learning. In the same way that parental engagement with children around shared play with toys facilitates children’s learning and exploration [49], we found that parent conversation (as measured around shared television viewing) could be similarly scaffolding, associated with higher expressions of child curiosity.

Prior research has demonstrated that children learn best in environments that are interactive, encouraging turn-taking, dialogic exchanges and intrinsically motivated questions [47, 50]. Our results similarly attest to this, but with an important consideration for children with socioeconomic disadvantage. While incremental increases in the frequency of parent conversation were associated with higher curiosity for *all* children, for children from under-resourced (i.e., *low SES*) environments, only parents who *often* engaged in conversation around shared television viewing had children whose curiosity scores were above the mean. Conversely, children from more-resourced environments (i.e., *high SES)* had curiosity scores above the mean even if parents *hardly ever* conversed when viewing television together. The “curiosity gap” between higher and lower SES children was greatest when parents “*never*” or “*hardly ever*” engaged in television-related conversation but was not observed when conversational exchanges occurred “*often*.” This suggests that for children from under-resourced environments, more frequent parent conversation may help enable the expression of curiosity. One implication is that parents from low SES environments might benefit from anticipatory guidance regarding the importance of dialogic (back and forth) conversation to promote inquisitiveness and learning. Such guidance may include interventions similar to “parent coaching” to facilitate conversational exchanges to promote early language development [51]. At present, because the dominant screen activity of low-income children involves television viewing [52], and because television viewing is essentially non-conversational and non-interactive [53], fostering opportunities for conversational exchanges around television viewing (in addition to other shared activities) may be one potential naturalistic intervention [47].

Our results also indicate that more frequent parent conversation was associated with parent reports of higher imagination at kindergarten. The topics eliciting a child’s curiosity are often related to a child’s idiosyncratic interests [54], and are revealed in the context of responsive, interactive exchanges [55]. Because we hypothesize that conversation around shared television viewing likely included pedagogical exchanges, (e.g., “*What* do you think is going on? *Why* do you think that happened?”), our results suggest the possibility that more frequent conversation (in all contexts, not just television viewing) can promote imaginative expression (one of the underpinnings of curiosity [56]) at kindergarten. Interventions to promote dialogic exchanges and language-rich caregiver-child interactions have been shown to be beneficial for early imagination and learning, and may be similarly promotive for early childhood curiosity [57, 58].

Our study had several strengths and limitations. Strengths include the use of a nationally representative sample which included a child behavior questionnaire from which we could derive a measure of curiosity, whose results are generalizable to the population. One limitation is that our study used parent self-reports to measure the amount of television viewing and parent conversation, and our curiosity factor was derived from a single parent-report behavioral measure at the kindergarten timepoint. As such, we acknowledge the potential bias and shared method variance associated with parent report measures. In addition, although a subsample analysis indicated that parents who engaged in more frequent television-related conversation were more likely to use elaborative language, there was no independent measure of non-television parent-child conversation for the entire sample, so we were unable to control for non-screen time language. Although there was a teacher-report of child behavior at kindergarten, it did not include all the “curiosity” items, so we could not examine curiosity across reporters. In addition, the dataset did not contain information regarding the content of the television programs watched, which is a potential confounder which we were not able to include in our analyses. We also acknowledge that while we found significant associations between the hours of television viewing, frequency of parent conversation and parent reports of curiosity, our effect sizes were small. Finally, while the ECLS-B is a rich dataset and among the only longitudinal cohorts from the United States, the data are older, and did not include measures of smartphones and other portable technologies on which television programming may be watched, along with more conversational media such as video-chatting, which is an additional limitation. Future research should consider examining these associations in relation to use of conversational and non-conversational digital media across screen platforms. Future research should also examine other features of curiosity that might help mitigate the poverty achievement gap [59], and consider other adaptive outcomes associated with early childhood curiosity [60]. Despite these limitations, we believe that our results have some important implications for caregivers and pediatricians.

## Conclusions

Our results suggest that more frequent parent-child conversations around television viewing (which may be a proxy for other conversational exchanges) are associated with *higher* curiosity, especially in children with socioeconomic disadvantage. This highlights the importance of parents engaging in reciprocal conversations around topics and experiences of mutual interest [47], and suggests the importance of finding opportunities foster conversational exchanges in the context of daily routines (e.g., even when watching television). Aligning with the American Academy of Pediatrics’ recommendations on media [61, 62], parents can be counseled on the value of parental instructive dialogue during television viewing (e.g. “active mediation”) [63], as an opportunity to promote inquiry [64]. Parent-child conversations that are guided by active mediation have been associated with more adaptive social-emotional development in young children, with a greater magnitude of effect in children from low-income families [65]. Our work extends this line of research and highlights the benefits of active mediation on early childhood *curiosity*. Because parent conversation around television viewing is likely related to parent conversation in the home, our results also suggest the importance of fostering opportunities for dialogic exchanges around all topics (not just television), especially for children from environments of socioeconomic disadvantage [27].

## Supporting information

S1 Appendix Adjusted associations of daily television viewing, parent conversation during shared television viewing and curiosity (Step 1 –main effects). (DOCX)

S2 Appendix Adjusted associations of daily television viewing, parent conversation during shared television viewing and curiosity, moderated by SES (Step 2- moderation results, prior to median split). (DOCX)

S3 Appendix Derived curiosity factor from the ECLS-B. (DOCX)
