# Supplementary material for: Daily television exposure, parent conversation during shared television viewing and socioeconomic status: Associations with curiosity at kindergarten
Source: PLoS One. 2021 Oct 28;16(10):e0258572. doi: 10.1371/journal.pone.0258572 (PMC8553096; doi:10.1371/journal.pone.0258572)
Supplement: S1 Appendix — (DOCX) [file pone.0258572.s001.docx]

**S1 Appendix:** **Adjusted Associations of Daily Television Viewing, Parent Conversation During Shared Television Viewing and Curiosity (Step 1 – Main Effects)**

**Step 1 – Main Effects** **B (SE) p____**

Hours of television viewing / day (linear term) -0.14 (0.05) .008

Main Effects

Hours of television viewing / day (quadratic term) 0.02 (0.01) .03

Parent Conversation during shared television viewing

Often 0.66 (0.11) <.001

Sometimes 0.42 (0.12) <.001

Hardly ever 0.31 (0.13) .02

Never (REF) ---- ----___

Socioeconomic Status 0.13 (.03) <.001

Maternal age -0.01 (0.003) .002

Race/Ethnicity

Covariates

Other 0.004 (0.09) .96

Asian -0.14 (0.06) .03

Hispanic 0.08 (0.06) .17

Black/Non-Hispanic -0.03 (0.05) .61

White (Ref) ---- ----

24-month Cognitive Development (Bayley-SFR) 0.01 (.002) <.001

Child’s age at kindergarten 0.01 (.004) .01

Child’s Sex (female) 0.15 (0.04) <.001

Ability to Delay Gratification 0.09 (0.04) .02

SOURCE: U.S. Department of Education, National Center for Education Statistics, Early Childhood Longitudinal Study, Birth Cohort. Selected years 2001-2007
