# Supplementary material for: Daily television exposure, parent conversation during shared television viewing and socioeconomic status: Associations with curiosity at kindergarten
Source: PLoS One. 2021 Oct 28;16(10):e0258572. doi: 10.1371/journal.pone.0258572 (PMC8553096; doi:10.1371/journal.pone.0258572)
Supplement: S2 Appendix — (DOCX) [file pone.0258572.s002.docx]

**S2 Appendix:** **Adjusted Associations of Daily Television Viewing, Parent Conversation During Shared Television Viewing and Curiosity, Moderated by SES (Step 2- Moderation Results, prior to median split)**

**Step 2 – Moderation by SES** **B (SE) p_____**

Hours of television viewing / day (linear term) -0.14 (0.05) .008

Main Effects

Hours of television viewing / day (quadratic term) 0.02 (0.01) .03

Parent Conversation during shared television viewing

Often 0.62 (0.10) <.001

Sometimes 0.39 (0.10) <.001

Hardly ever 0.28 (0.11) .01

Never (REF) ---- ----

_Socioeconomic Status 0.53 (.13) <.001___

Moderation x SES

SES X Parent Conversation during shared television viewing

SES x Often -0.50 (0.12) <.001

SES x Sometimes -0.34 (0.12) .008

SES x Hardly ever - 0.27 (0.14) .06

___ SES x Never (REF) ---- ----_____

Maternal age -0.01 (0.003) .002

Race/Ethnicity

Other -0.003 (0.09) .97

Covariates

Asian -0.14 (0.06) .03

Hispanic 0.09 (0.06) .12

Black/Non-Hispanic -0.01 (0.05) .77

White (Ref) ---- ----

24-month Cognitive Development (Bayley-SFR) 0.01 (.002) <.001

Child’s age at kindergarten 0.01 (.004) .01

Child’s Sex (female) 0.15 (0.04) <.001

___Ability to Delay Gratification 0.09 (0.04) .02______

SOURCE: U.S. Department of Education, National Center for Education Statistics, Early Childhood Longitudinal Study, Birth Cohort. Selected years 2001-2007
