# Supplementary material for: Daily television exposure, parent conversation during shared television viewing and socioeconomic status: Associations with curiosity at kindergarten
Source: PLoS One. 2021 Oct 28;16(10):e0258572. doi: 10.1371/journal.pone.0258572 (PMC8553096; doi:10.1371/journal.pone.0258572)
Supplement: S3 Appendix — (DOCX) [file pone.0258572.s003.docx]

**S3 Appendix:** **Derived Curiosity Factor from the ECLS-B**

Question Items in Derived Curiosity Factor______ Loading Coefficient________________

Likes to try new things 0.71

Shows imagination in work and play 0.58

Shows eagerness to learn new things 0.59

Easily adjusts to a new situation 0.56

______________________________________________________________________________

Cronbach’s alpha: α = 0.70

Adjusted GFI = 0.91

Standardized RMR (SRMR) = 0.047

SOURCE: U.S. Department of Education, National Center for Education Statistics, Early Childhood Longitudinal Study, Birth Cohort. Selected years 2001-2007
